# Delayed Diagnosis of an Adult Epiglottic Abscess: A Case Report

**DOI:** 10.7759/cureus.95152

**Published:** 2025-10-22

**Authors:** Sami Sebeih, Rama A Alraheili

**Affiliations:** 1 Emergency Medicine, Prince Mohammed Bin Abdulaziz Hospital, Al Madinah, SAU

**Keywords:** airway obstruction, emergency medicine, epiglottic abscess, epiglottitis, revisit

## Abstract

Distinguishing acute epiglottitis from other benign causes of sore throat can be challenging in the adult age group, and failure to do so can lead to an airway-related death. We report the case of a 65-year-old man with type 2 diabetes who initially presented with sore throat, odynophagia, and fever, and was twice discharged with presumed pharyngitis after outpatient and emergency department evaluations. Within 24 hours, he returned with worsening odynophagia and inability to swallow medications. Despite stable vital signs and absence of stridor and drooling, laboratory testing revealed leukocytosis and markedly elevated inflammatory markers. Lateral neck radiography demonstrated the thumbprint sign, and computed tomography confirmed a 28×24×18 mm periepiglottic abscess. Flexible laryngoscopy revealed severe hypopharyngeal swelling with an epiglottic abscess. He underwent controlled awake fiberoptic intubation followed by surgical drainage, intravenous antibiotics, corticosteroids, and supportive care, with full recovery and discharge after four days. This case highlights that persistent sore throat and odynophagia refractory to initial therapy should raise suspicion for epiglottitis or an epiglottic abscess, even in well-appearing adults. Early imaging, mobilization of airway experts, and proactive airway planning are critical for preventing catastrophic airway loss.

## Introduction

Epiglottitis in adults is an uncommon but potentially life-threatening condition that requires urgent recognition and airway management. Although widespread *Haemophilus influenzae* type B vaccination has reduced pediatric incidence, the proportion of adult cases has been rising [1]. The clinical presentation is often subtle, with sore throat, odynophagia, and dysphonia mimicking benign pharyngitis, which may delay diagnosis [2]. Unlike children, adults frequently lack dramatic airway obstruction, and up to 44% may have a normal oropharyngeal examination, increasing the risk of misdiagnosis [3]. One of the most serious complications is an epiglottic abscess, reported in up to 24% of cases, which significantly raises the likelihood of airway compromise [4,5]. Failure to recognize epiglottitis or to mobilize airway experts promptly may result in catastrophic outcomes. We present a case of delayed diagnosis of an epiglottic abscess in an older adult initially treated for pharyngitis, emphasizing the need for a high index of suspicion in patients with persistent or worsening upper airway symptoms.

## Case presentation

In late December 2024, a 65-year-old male patient presented to our emergency department (ED) for a sore throat that had started three days prior to his initial presentation. He endorsed painful swallowing as well as fever. He denied cough, rhinorrhea, shortness of breath, and chest pain. The patient had a medical history of type 2 diabetes for which he was taking oral medications. He had no known allergies, was otherwise healthy, and had no prior history of airway disease. Earlier in his course, before visiting our ED, he had gone to a private healthcare facility and was discharged as a case of acute pharyngitis with oral antibiotics and antipyretic; however, he failed to improve. He went again to the private clinic where he received intravenous amoxicillin and acetaminophen and was discharged. His examination revealed a well but dehydrated patient, with low-grade fever (37.6 degrees) and mild tachycardia (107 bpm). His blood pressure was normal, and he did not have tachypnea or respiratory distress, and maintained normal saturation in room air. His voice was raspy. The throat examination was significant for mild pharyngeal congestion without any abnormality in the tonsils. The patient was discharged as a case of acute pharyngitis with oral antibiotics and dexamethasone, keeping with the initial impression of acute pharyngitis.

**Figure 1:**
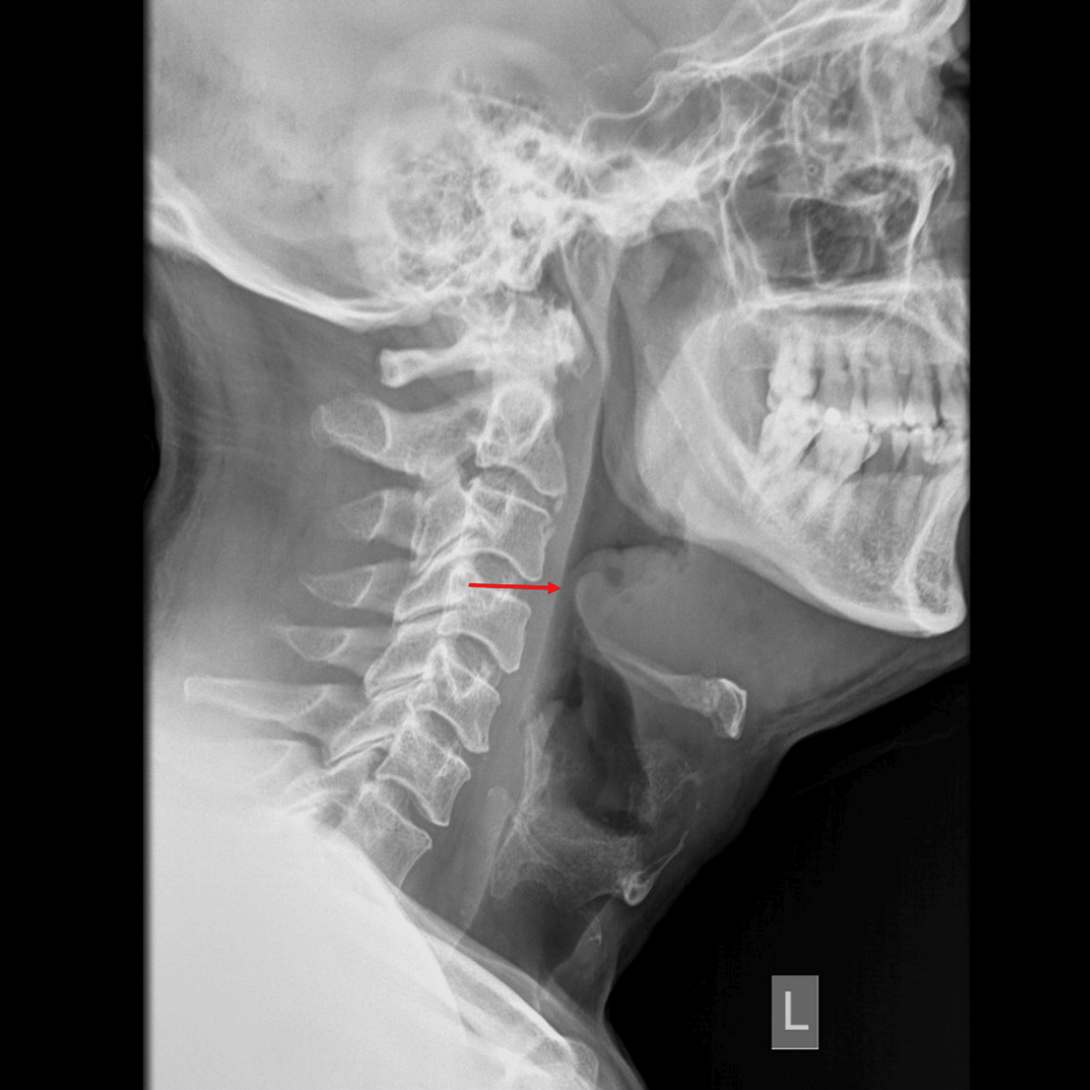
Soft tissue neck X-ray showing the enlarged epiglottis (thumbprint sign)

Within 24 hours, he revisited our ED for more severe painful swallowing and difficulty swallowing tablets, and he expected to receive IV medications. Vital signs showed a fever of 38 degrees as well as a tachycardia of 103 bpm. He looked well with no tachypnea, drooling, or stridor, and he tolerated the supine position comfortably. Laboratory testing revealed a white blood cell (WBC) count of 14.1x10^9^/L (reference: 4-11 × 10^9^/L), and an elevated C-reactive protein level of 256.9 mg/L (reference: <5 mg/L). Urea and electrolyte levels were within normal limits. A lateral soft tissue X-ray showed an edematous and enlarged epiglottis projecting into the air column, classically described as the "thumbprint sign" (Figure 1). 

**Figure 2:**
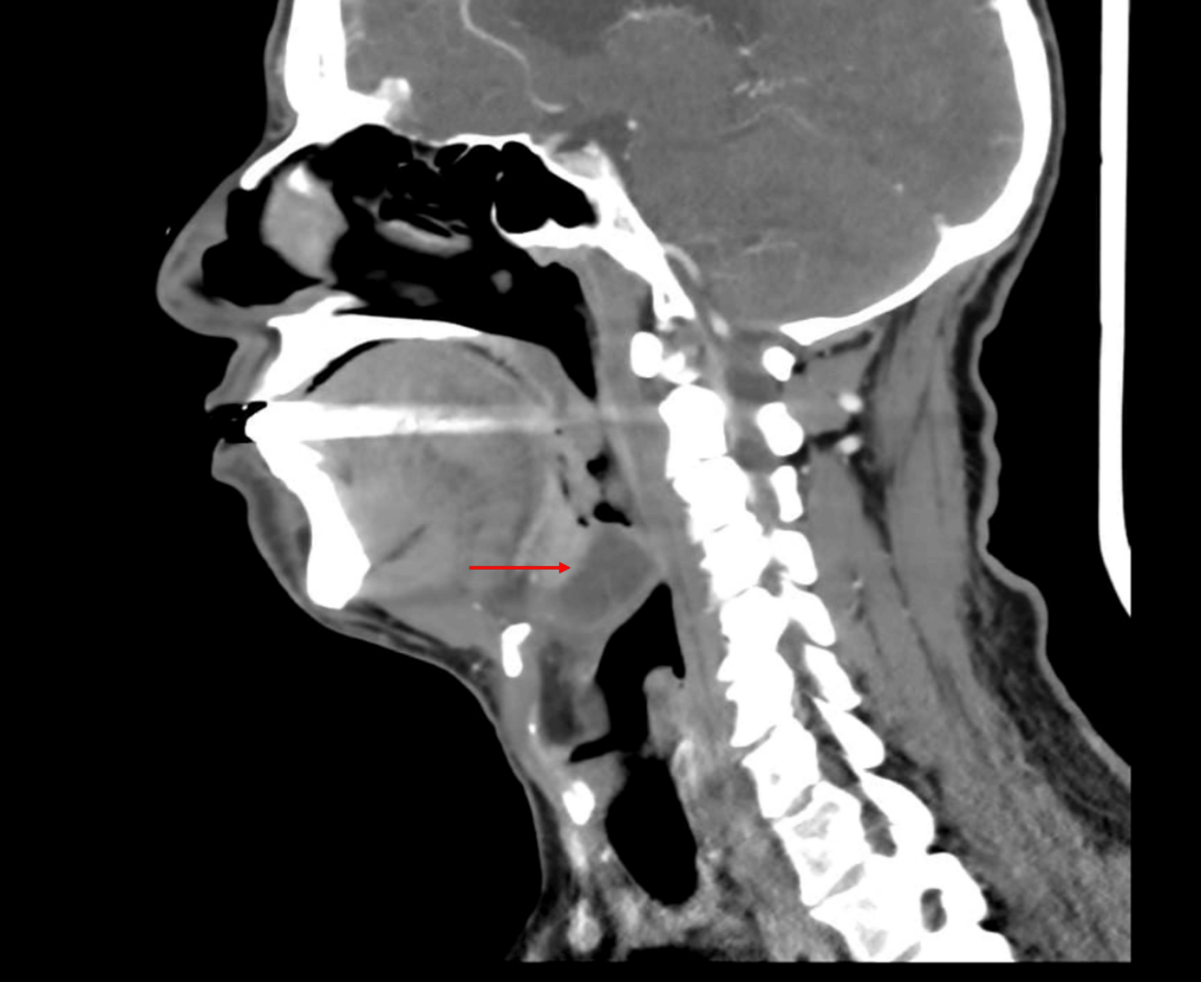
CT with contrast showing epiglottitis and epiglottic abscess

**Figure 3:**
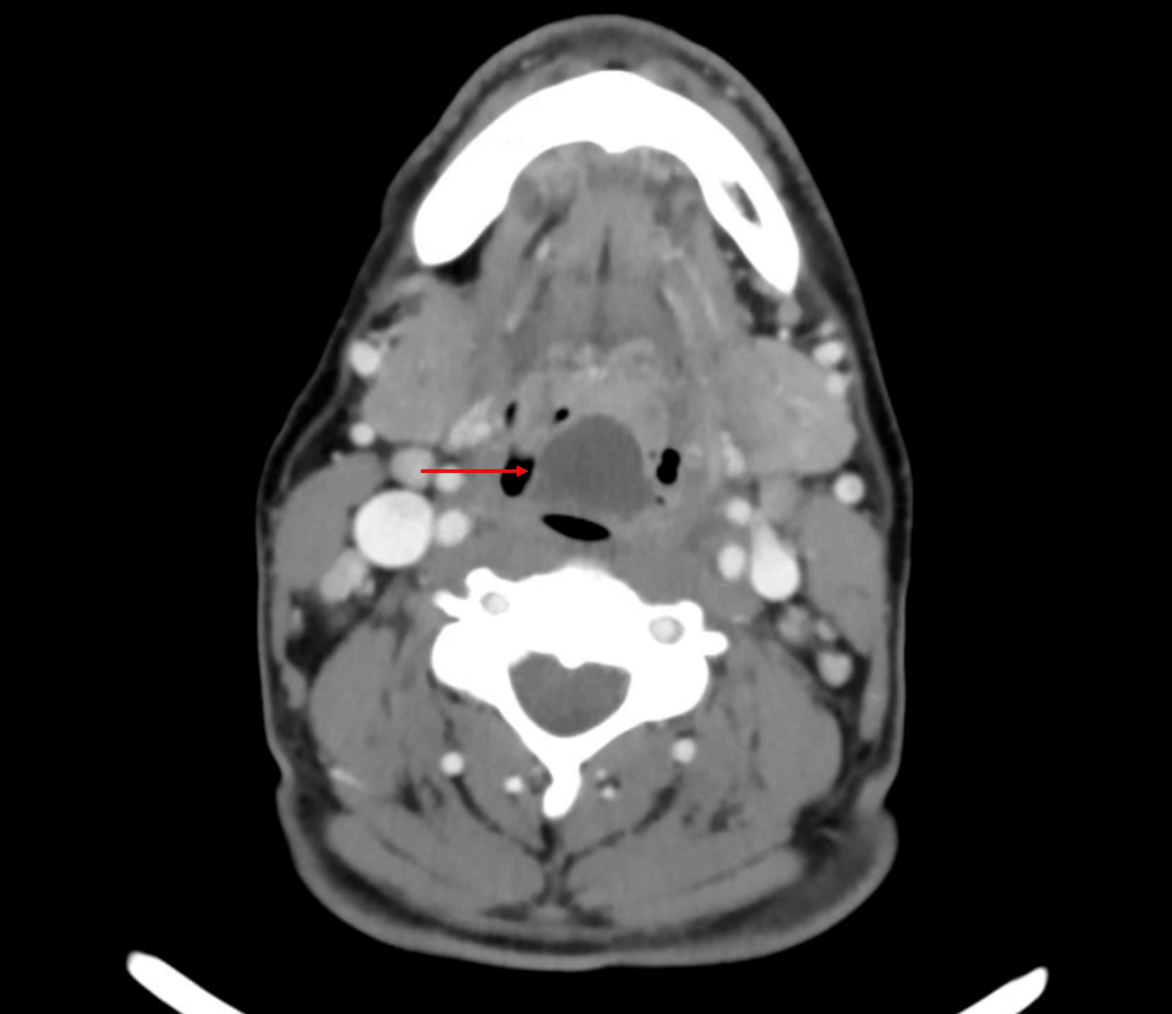
Severe airway obstruction caused by the epiglottic abscess

A computed tomography (CT) scan of the neck with contrast was obtained to confirm the diagnosis and to rule out complications. It revealed a well-defined collection, 28x24x18 mm in size, with peripheral enhancement in the upper portion of epiglottis suggestive of epiglottitis and epiglottic abscess (Figures 2, 3). 

The case was discussed immediately with the on-call ear, nose and throat physician. A flexible laryngoscopy showed severe hypopharyngeal swelling and an abscess of 3x2 cm occupying the lingual surface of the epiglottis pushing the epiglottis forward, and making it difficult to visualize the larynx. The patient received STAT IV 1 g ceftriaxone, IV 600 mg clindamycin, IV 8 mg dexamethasone alongside with antipyretics and hydration. Though he appeared comfortable without any sign of respiratory distress, the patient was taken to the operating room (OR) immediately for emergency controlled awake fiberoptic nasotracheal intubation. Under general anesthesia, an incision and drainage was done and a large amount of pus was suctioned and sent for culture and sensitivity. The patient tolerated the procedure well without any complications. He was extubated in the OR and admitted in the intensive care unit (ICU) for observation. He received IV 600 mg clindamycin every six hours, IV 1 g ceftriaxone every 12 hours and had an uneventful stay without desaturation or respiratory distress. He tolerated oral intake, and his inflammatory markers and WBC count showed a downward trend, and culture showed anaerobic gram-negative bacilli. He was discharged after four days on oral cefuroxime 500 mg every 12 hours and metronidazole every eight hours for seven days. The patient had a post-discharge follow up and was asymptomatic with normal laryngoscopic examination. 

## Discussion

Acute pharyngitis with progressively worsening upper airway symptoms such as dysphagia, odynophagia, and hoarseness that are refractory to conservative therapy should prompt consideration of life-threatening conditions that mimic acute pharyngitis [6]. Acute epiglottitis is one such condition, and if unrecognized, may lead to fatal airway obstruction [7]. Despite its rarity, epiglottitis remains a recognized cause of acute airway compromise, with mortality reported to be up to 20% [8]. The incidence of adult epiglottitis ranges from 0.88 to 3.1 per 100,000 annually [1].

An epiglottic abscess is a frequent complication. A multicenter study found abscesses in 22% of patients with suspected epiglottitis [4], a retrospective series of 140 adults reported 19.3% [5], and a 15-year cohort of 118 cases found 24% [1]. However, another study of 308 patients reported a lower rate of 7% [9]. This variability may reflect differences in the routine use of CT imaging. Overall, the incidence of epiglottic abscess appears to be less than 25%, though it is generally reported as a complication of epiglottitis rather than as an independent epidemiologic entity.

Distinguishing acute pharyngitis from epiglottitis requires attention to key clinical differences. Pharyngitis, viral or bacterial, typically presents with sore throat, fever, pharyngeal erythema, and may include tonsillar exudates or cervical lymphadenopathy. Viral etiologies often include cough, rhinorrhea, ocular irritation and oropharyngeal ulcers or vesicles. Importantly, pharyngitis rarely causes severe dysphagia, odynophagia, drooling, or airway compromise [10].

Epiglottitis should be suspected in patients with rapid-onset sore throat and odynophagia disproportionate to exam findings. Sore throat is reported in 88-100% of cases, dysphagia in 71-100%, odynophagia in 58-100%, muffled voice in 50-56%, while fever and tachycardia occur in about half the cases [11,12]. Stridor, though less common, is highly specific for impending airway loss, and drooling, though more common in children, is a strong diagnostic clue in adults [13].

Compared to pediatric patients, airway obstruction in adults is less dramatic due to a broader, more rigid epiglottis [14]. Oropharyngeal findings are frequently subtle or absent, as up to 44% of adults have a normal oropharyngeal exam [3]. When abnormalities are present, findings may include mild erythema or base-of-tongue swelling, but they are nonspecific [15,16]. Thus, laryngoscopy is required for confirmation, and even when an abscess complicates epiglottitis, a swelling on oropharyngeal inspection is uncommon [17].

Certain predictors that suggest epiglottic abscesses include muffled voice (odds ratio ~2.6), respiratory distress (associated with severe disease), moderate-to-severe epiglottic edema (odds ratio ~3.9), and pre-existing epiglottic cysts [5,18]. Age, comorbidities (including diabetes), obesity, tobacco use, and seasonality are not consistently associated [13].

“The eyes do not see what the mind does not know.” Likewise, knowledge of the usual course of adult epiglottitis as well as maintaining a high index of suspicion are crucial to diagnosis and preventing death.

Lateral neck radiography (LNR), performed with the patient erect and neck slightly extended, is a basic, widely available tool for preliminary diagnosis in stable patients, as in our case, but is not recommended for routine use in all upper respiratory infections [19]. The classical “thumbprint sign” demonstrates a thickened, swollen epiglottis and has moderate-to-high sensitivity (66.7-100%) and specificity (89.3%) [20]. Additional findings include the vallecula sign (absent vallecular air pocket), aryepiglottic fold swelling, prevertebral soft tissue thickening, and dilatation of the hypopharyngeal air column [21].

One study comparing LNR with laryngoscopy reported sensitivity 81%, specificity 85.7%, positive predictive value (PPV) 58.6%, and negative predictive value (NPV) 94.7%, with a 0.22 negative likelihood ratio, indicating LNR cannot exclude epiglottitis when suspicion is high [22]. False-negative rates up to 32% have been reported, especially with prior antibiotic use [23]. To improve objectivity, epiglottic width >8 mm and aryepiglottic fold width >7 mm were proposed, but data were limited (n=13) [24]. Later studies refined cutoffs: epiglottic width >6.3 mm (sensitivity 75.8%, specificity 97.8%) and >5.5 mm (sensitivity 96.2%, specificity 100%) [23,24]. A large case-control study (260 patients) suggested epiglottic width >5 mm (sensitivity 96.2%, specificity 98.2%) and aryepiglottic fold width >6.6 mm (sensitivity 86.5%, specificity 78.8%) [25].

Contrasted CT demonstrates 88-100% sensitivity and 96% specificity, while also identifying abscesses and alternative diagnoses such as lingual tonsillitis, peritonsillar or deep neck abscess, and foreign bodies [15]. However, supine positioning increases airway risk, so a tolerance trial is essential prior to imaging [8].

Point-of-care ultrasound (POCUS) has emerged as a noninvasive bedside option. A healthy epiglottis appears as a thin hypoechoic structure near the hyoid. In epiglottitis, thickening (>3.2 mm at the lateral edge) and the “alphabet P sign” (hypoechoic halo around the epiglottis with hyoid acoustic shadow forming a P) are characteristic findings [26,27]. Although not yet standard, POCUS is increasingly recognized as a useful adjunct when traditional imaging is unsafe or impractical.

## Conclusions

Adult epiglottitis and its complications, particularly epiglottic abscess, can masquerade as simple pharyngitis and progress rapidly to life-threatening airway compromise if overlooked. This case highlights the importance of “thinking twice” when symptoms worsen despite conservative therapy. Clinicians must maintain a high index of suspicion in adults with disproportionate odynophagia, muffled voice, or dysphagia, even when oropharyngeal findings are subtle.

Timely diagnosis hinges on the judicious use of diagnostic tools. While lateral neck radiography may reveal the classical “thumbprint sign,” it cannot reliably exclude disease. Contrast-enhanced CT remains highly sensitive and specific for epiglottitis and its complications, though airway stability must be ensured before imaging. POCUS offers a promising, noninvasive bedside alternative, especially in unstable patients.

The take-home message is that when adult patients return with worsening pharyngitis symptoms, physicians must prioritize airway assessment and consider epiglottitis. Diagnosis should be guided by a stepwise approach using clinical suspicion supported by radiography, CT, or bedside ultrasound. Early recognition and appropriate airway management remain the cornerstone options for preventing preventable mortality.
